# A Rare Case of Lumbar Region Diastematomyelia in a 25-year-old Woman With Lower Back Pain

**DOI:** 10.7759/cureus.51796

**Published:** 2024-01-07

**Authors:** Vladislav Velchev, Bogomil Iliev, Petar-Preslav Petrov, Ilko Ilyov, Plamen Penchev

**Affiliations:** 1 Faculty of Medicine, Medical University of Plovdiv, Plovdiv, BGR; 2 Department of Neurosurgery, Medical University of Varna, Varna, BGR; 3 Clinic of Neurosurgery, University Hospital “Saint Marina”, Varna, BGR; 4 Department of Anatomy, Histology and Embryology, Medical University of Plovdiv, Plovdiv, BGR

**Keywords:** diastematomyelia, low back pain, scoliosis, rare case, visual analog scale (vas)

## Abstract

Diastematomyelia is an infrequent congenital anomaly that anatomically presents with a longitudinal division of the spinal cord at the level of one or several sections. It is generally diagnosed and treated in children. We present a 25-year-old female patient who has entered the Neurosurgery clinic after a traumatic experience with severe low back pain evaluated with 7 points on the visual analog scale (VAS). Magnetic resonance imaging (MRI) of the lumbar area discovered evidence for diastematomyelia type 1 at levels L3-L5 with deformations in L4 and L5 vertebrae and mild scoliosis. Because of the lack of neurological deficiency, the patient’s treatment was conservative and included a 10-day intake of nonsteroid anti-inflammatory drugs (NSAIDs) in combination with myorelaxant and physiotherapy. On the 15^th^ day, the patient was evaluated with 2 points on VAS. Neurological follow-up examinations were conducted on the third and the sixth month and the patient was evaluated with VAS 1, which proves that conservative treatment is successful for diastematomyelia in adults without neurological deficiency.

## Introduction

Diastematomyelia is an infrequent congenital anomaly that anatomically presents with a longitudinal division of the spinal cord at the level of several vertebrae. The hemicords are relatively equal in size, shape, and structure, composed of ventral and dorsal horns and neural roots, as well as a central canal. Diastematomyelia is a result of spina bifida, a neural tube malformation causing a gap in the spine [1]. The age groups mostly affected by this disorder are children and adolescents. Few cases of adults with such a condition have been reported. The frequent clinical manifestations involve scoliosis, musculoskeletal asymmetry, weakness in the legs, and dorsal skin marks. Nearly half of the cases express cutaneous abnormalities on the back, such as a hairy patch, dimples, pigmented areas, haemangioma, and subcutaneous mass [2]. Neurological symptoms of the condition are indefinite and do not always cause health issues. Such are sensitivity abnormalities like a feeling of pain, or motor weakness of the limbs.

By the latest statistics, diastematomyelia appears in two to four in 1000 newborns therefore it is an extremely rare spinal cord disorder [3]. It is most presented in the lumbar sections of the spinal cord, although such malformations in the cervical and thoracic areas have also been reported. Two types of diastematomyelia have been differentiated. Type 1 patients have a double neural tube and account for approximately 40% of the cases. The split of the spinal cord could, in some cases, be accompanied by the formation of a cartilaginous or bony spur between the hemicords. It commonly causes neurological symptoms in early childhood due to cord tethering. Type 2 patients have a single neural tube and are usually asymptomatic [1].

The aim of this study is to report a rare case of an MRI-diagnosed diastematomyelia of the lumbar region in a 25-year-old female patient with low back pain and no neurological deficiency and to evaluate the symptomatic manifestations, background, and treatment alternatives for patients with late diagnosis.

## Case presentation

**Figure 1:**
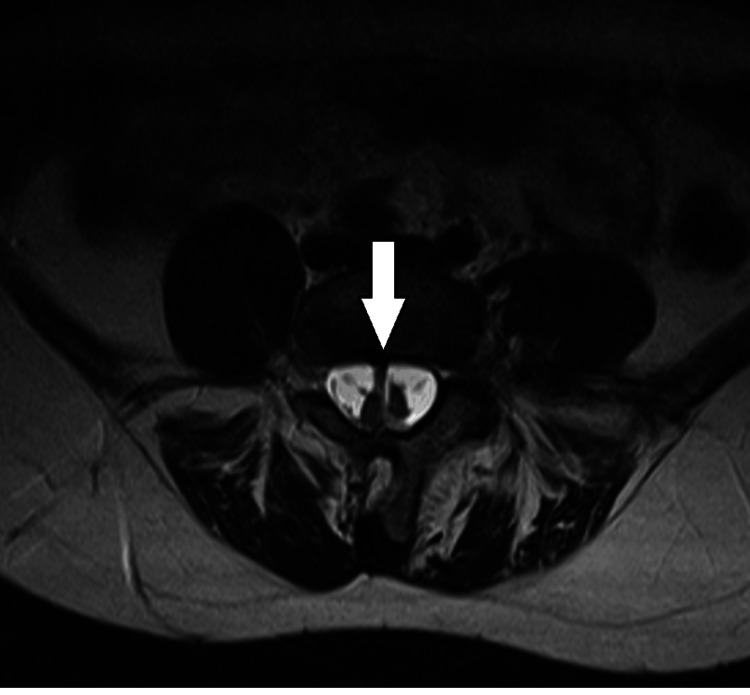
MRI of L4-L5 diastematomyelia (axial plane) The hemicords have been split by a bony spur. The arrow points at the bony spur between the hemicords.

**Figure 2:**
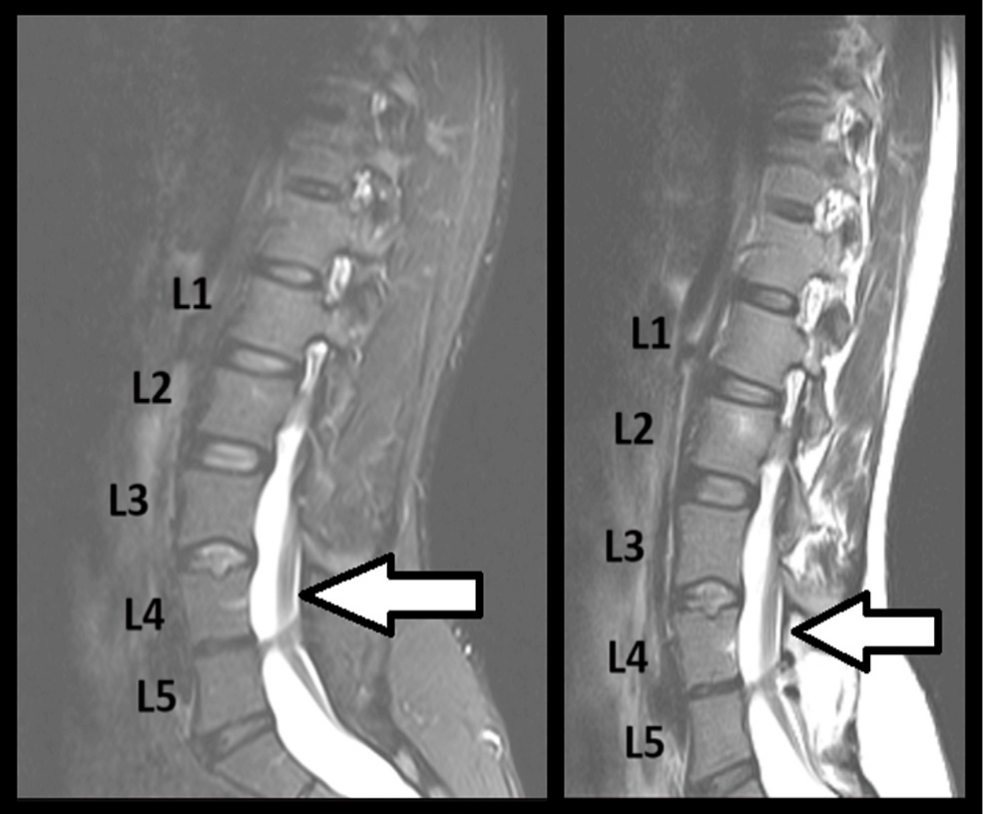
MRI of L4-L5 diastematomyelia (sagittal plane) The hemicords have been split by a bony spur at the level L4-L5. Deformations of L4 and L5 vertebrae are visible. Note the bony spur dividing the hemicords (arrows).

**Figure 3:**
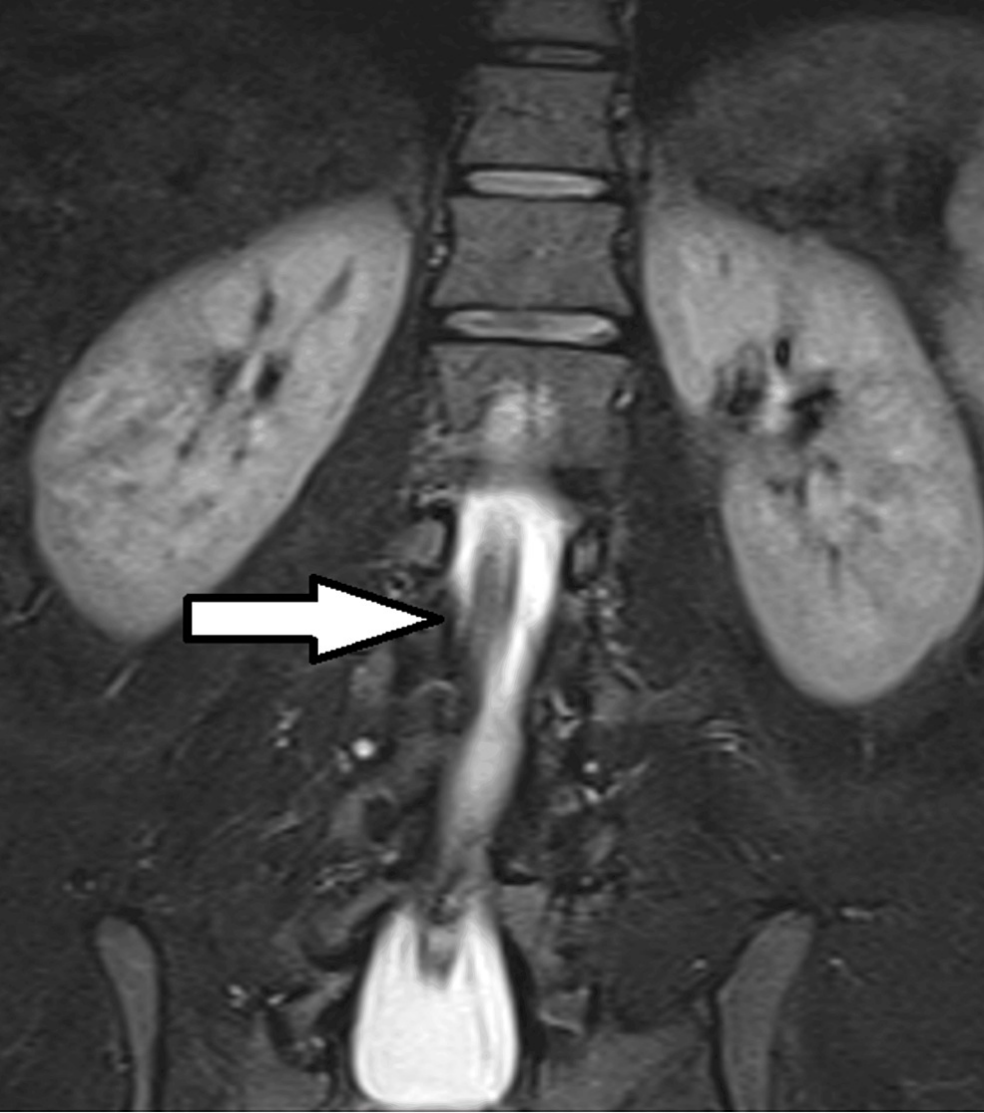
MRI of diastematomyelia and mild scoliosis in L4-L5 sections of the spinal cord (coronal plane) Note the bony spur dividing the hemicords (arrow).

**Figure 4:**
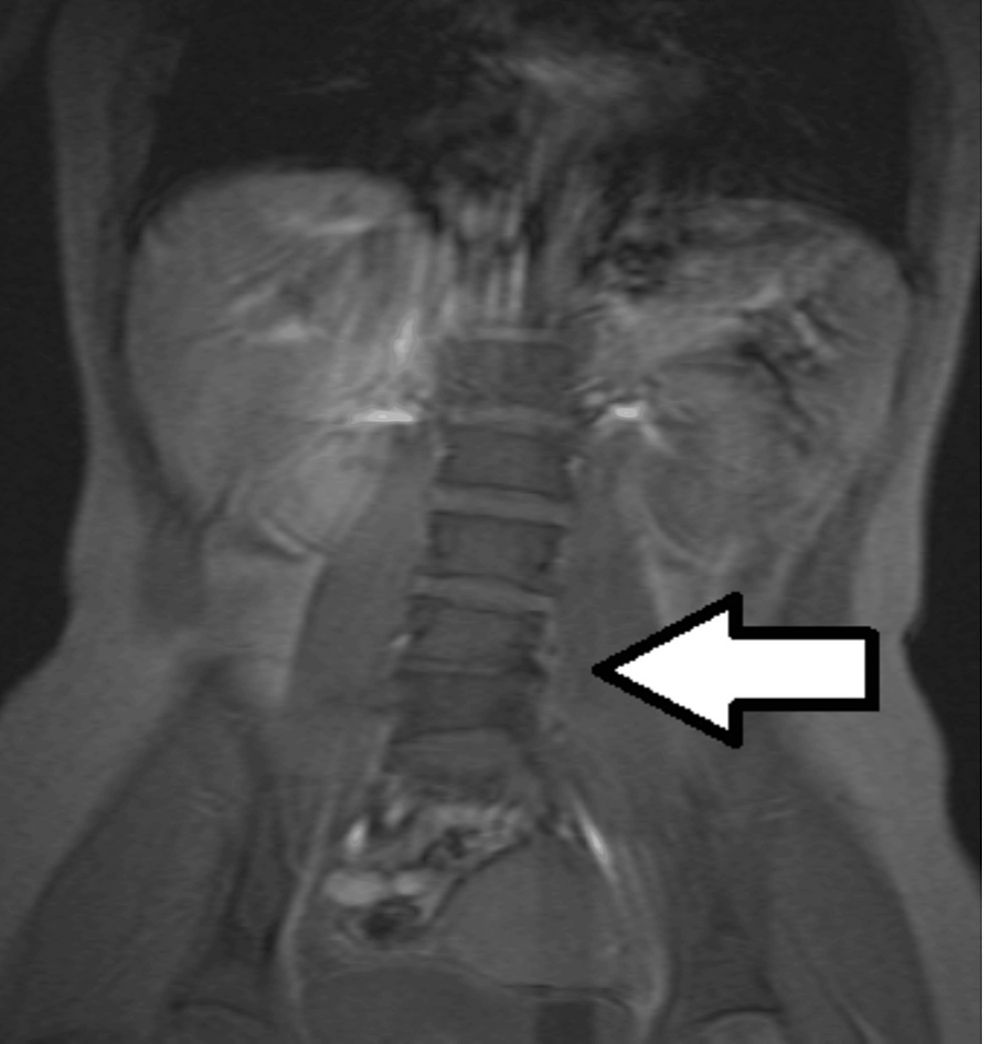
MRI of mild scoliosis in L4-L5 region Note the mild scoliosis (arrow).

We present a 25-year-old female patient who has entered the neurosurgery clinic with severe low back pain after a traumatic experience. The neurological status presents only with a vertebral syndrome in the lumbar region evaluated with VAS 7. Through a comprehensive examination of the local status, discrete spots on the skin were observed. MRI of the lumbar area discovered evidence for diastematomyelia type 1 at levels L3-L5 (Figures 1-3) with deformations in L4 and L5 vertebrae (Figure 2) and mild scoliosis (Figure 4).

Because of the lack of neurological deficiency, the patient’s treatment was conservative. The non-steroidal anti-inflammatory drug (NSAID) dexketoprofen 2 ml/ampule was administered intravenously for five days, morning and evening, with a bank of physiological serum and pantoprazole 15 mg in a dosage of 2x1 capsule. After discharge on the fifth day, the treatment continued with meloxicam 7.5 mg in a dosage of 2x1 tablets (tab). Throughout the course, a muscle relaxant thiocolchicoside 4 mg in a dosage of 2x1 tab, was administered.

Physiotherapy started on the seventh day after the acute complaints subsided and mainly included procedures with ultrasound, magnetic therapy, and balneotherapy with thermal mineral water. After the hospital treatment, the pain in the lower back was controlled and the VAS was 4 points. After completing the entire course of treatment on the 15th day, the patient was evaluated with VAS 2. Neurological follow-up examinations were conducted on the third and the sixth month and the patient was evaluated with VAS 1.

## Discussion

Symptoms and signs of lumbar diastematomyelia

Pain in diastematomyelia may be due to the spinal cord pathology, the compression of the spinal nerves, or the formation of an abnormal cartilaginous obstruction between the two hemicords. Clinical manifestations of the abnormality result from a defect in the anatomical structure of the spinal cord and its efferent neurons in regions L1-L5 and S1-S4. The 25-year-old female patient in this case similarly experienced low back pain and her hemicords have been divided by a bony spur but has no signs of further motor or sensory neurological issues.

Diastematomyelia is a kind of spinal dysraphism. Such disorders are clinically presented with the following signs: skin-covered lesion involving two or more vertebrae, visible only on radiography; hair tuft, lipoma, or other cutaneous features [4]. Our patient exhibits skin spots visible only after a comprehensive review.

In relation to the anatomical specifications and common symptoms of the disorder, two variants of diastematomyelia have been identified. Type 1 and type 2 diastematomyelia have the following characteristics presented in Table 1 [5]. MRI of our patient and the neurological examination have discovered evidence for diastematomyelia type 1.

**Table 1:** Classification of diastematomyelia

	Diastematomyelia type 1	Diastematomyelia type 2
Incidence	40% – 50%	50% – 60%
Neural tubes	double	single
Presence of spur	yes	no
Symptoms	Malsegmentation anomalies, scoliosis, pain, etc.	Usually asymptomatic

Demographic statistics

Around 80% of diastematomyelia cases are identified in childhood and express clinical manifestations before the age of five [6]. The case presented in this report regarding adolescents is extremely rare as the malformation has continued to develop to a later period of life. A few examples of spinal cord division in adulthood have been reported [6]. Therefore, symptoms are yet unclear and treatment techniques are in progression.

For patients with mild symptoms, the disorder could remain undiagnosed and patients’ complaints to be postponed. In a study covering 138 cases of diastematomyelia, Cheng et al. discovered that 87% of their patients were under the age of 16 and only 13% were above that line [1]. In this study, 81% of the patients exhibited neurological symptoms, suggesting grounds for the necessity for surgical intervention; 19% of the cases reviewed had no neurological deficit and mild complaints therefore not treated surgically [1]. It is noticeable that 90% of the patients are of female gender. The rare case presented in this report of diastematomyelia type I diagnosed at the age of 25 years is an example of the late manifestation of the symptoms as a result of physical exertion and lifting heavy objects.

Surgery

When patients diagnosed with diastematomyelia experience neurological issues, surgery is the only way of treating the disorder [7]. Pavlova et al. have also reported 27 patients with diastematomyelia who underwent surgeries. The mean age of the diagnosed was approximately nine years. The intervention includes decompression of the neural elements, excision of the tissue between the hemicords, and reconstruction of the doubled dural sacs [8].

The division of the spinal cord could, in some cases, be accompanied by the formation of a bony or cartilaginous gland between the hemicords. This requires a surgical intervention in which the formation is removed with precision [1]. The intervention includes decompression of the neural elements, excision of the tissue between the hemicords and reconstruction of the doubled dural sacs [8]. MRI of the patient presented in this case also discovered the presence of a bony spur, which should be removed surgically if conservative treatment fails or the condition worsens over time.

Embryonic background

The origin of diastematomyelia is being researched and is proven to be rooted in a defective secondary neurulation of the embryo. In 2023, Arbelo-Pérez et al. documented a case of such neural tube malformation and diagnosed it prenatally. Ultrasound examination has discovered the disorder, and the MRI scan has confirmed the diagnosis [9].

Conservative treatment

For patients with no neurological deficiency, conservative treatment is enough to relieve the symptoms. A combination of pharmacotherapy and physiotherapy has the potential to better the patient’s condition [10]. An NSAID and a myorelaxant have been prescribed to the 25-year-old woman with diastematomyelia. In a recent comprehensive review of treatment, Csiba et al. have concluded that the use of muscle relaxants (including tolperisone, tizanidine, thiocolchicoside, and baclofen) provides a fast pain relief effect in patients with acute low back pain [11].

Miller et al. claimed that treating patients with good neurological status should be physiotherapy and follow-up. This method has also been chosen for the treatment of the patient reported in this article. The purpose of the physiotherapy is to ease the pain in the affected regions [12]. This conservative way of treatment is a good alternative to surgical intervention but is not secure enough and requires a long follow-up and frequent examinations. Physiotherapy is a temporary solution to ease the symptoms, yet the malformation can still affect the motor and sensory reflexes [13]. Should it not make a positive impact, surgery is highly recommended and is the only method of complete recovery.

Stanescu et al. share that after 10 sessions of rehabilitation involving kinesitherapy, their patient has improved significantly [14]. Our patient’s results are positive as well, but follow-up examinations are required as the condition may develop and cause further neurological complications. Therefore, surgery is not the only method for treating diastematomyelia, and conservative treatment is a rational alternative for mild cases.

## Conclusions

Diastematomyelia is a rare congenital disease usually diagnosed and treated in children. This rare case of diastematomyelia in a 25-year-old demonstrates the complexity and management of this anomaly in adulthood. For such cases with a lack of neurological deficiency, conservative treatment with NSAIDs and a myorelaxant followed by physiotherapy is an effective method for temporary relief of the clinical manifestations. VAS demonstrated significant improvement in this case, though follow-up examinations are compulsory to avoid further neurological complications. Should the treatment be insufficient and the symptoms return, surgical intervention will be needed in the future.
